# The genetic control of immunity to *Plasmodium* infection

**DOI:** 10.1186/s12865-015-0078-z

**Published:** 2015-03-26

**Authors:** Audrey V Grant, Christian Roussilhon, Richard Paul, Anavaj Sakuntabhai

**Affiliations:** Unité de la Génétique Fonctionnelle des Maladies Infectieuses, Institut Pasteur, Paris, France; Centre National de la Recherche Scientifique, URA3012 Paris, France

**Keywords:** Malaria, Genome-wide association study, Parasitemia, Humoral immunity

## Abstract

**Background:**

Malaria remains a major worldwide public health problem with ~207 million cases and ~627,000 deaths per year, mainly affecting children under five years of age in Africa. Recent efforts at elaborating a genetic architecture of malaria have focused on severe malaria, leading to the identification of two new genes and confirmation of previously known variants in *HBB*, *ABO* and *G6PD,* by exploring the whole human genome in genome-wide association (GWA) studies. Molecular pathways controlling phenotypes representing effectiveness of host immunity, notably parasitemia and IgG levels, are of particular interest given the current lack of an efficacious vaccine and the need for new treatment options.

**Results:**

We propose a global causal framework of malaria phenotypes implicating progression from the initial infection with *Plasmodium spp.* to the development of the infection through liver and blood-stage multiplication cycles (parasitemia as a quantitative trait), to clinical malaria attack, and finally to severe malaria. Genetic polymorphism may control any of these stages, such that preceding stages act as mediators of subsequent stages. A biomarker of humoral immunity, IgG levels, can also be integrated into the framework, potentially mediating the impact of polymorphism by limiting parasitemia levels. Current knowledge of the genetic basis of parasitemia levels and IgG levels is reviewed through key examples including the hemoglobinopathies, showing that the protective effect of *HBB* variants on malaria clinical phenotypes may partially be mediated through parasitemia and cytophilic IgG levels. Another example is the IgG receptor FcγRIIa, encoded by *FCGR2A,* such that H131 homozygotes displayed higher IgG2 levels and were protective against high parasitemia and onset of malaria symptoms as shown in a causal diagram.

**Conclusions:**

We thus underline the value of parasitemia and IgG levels as phenotypes in the understanding of the human genetic architecture of malaria, and the need for applying GWA approaches to these phenotypes.

## Introduction

Malaria remains a major worldwide public health problem in spite of numerous international control efforts with ~207 million cases in 2012 and ~627,000 deaths per year, mainly affecting children under five years of age in Africa (http://www.who.int/en/). Malaria in humans is caused by infection with the protozoan *Plasmodium spp.*, *falciparum*, *vivax*, *ovale, malariae* or *knowlesi. Plasmodium falciparum* is responsible for the most severe form of malaria. No efficacious malaria vaccine exists, and current treatment options are threatened by the spread of drug resistant parasite strains. The malaria parasite has a complex lifecycle, initiated when an infected female *Anopheles* mosquito takes a bloodmeal, during which sporozoites are injected into the bloodstream and then invade liver cells. After asexual replication in the liver, merozoites are released into the blood and further asexual multiplication occurs in red blood cells. Merozoites mature into trophozoites, and a small proportion develop into female and male gametocytes, infectious to the mosquito, thereby completing the cycle. In endemic regions, it is expected that most individuals will become infected at some time, and a small fraction go on to develop life-threatening severe malaria with complications such as cerebral malaria, respiratory distress, or anemia. Detectable parasitemia is used to diagnose clinical malaria and high parasitemia to diagnose severe malaria (http://www.who.int/en/). The search for human genetic factors that influence parasitemia levels and other markers of anti-malarial host immunity could lead to novel molecular strategies for vaccine and treatment development, and malaria control. We therefore explore the potential of quantitative measures of malaria infection, including parasitemia and the biomarker of humoral host immunity, IgG levels, in the search for human genetic factors impacting on malaria, in this review.

## Review

### Genome-wide association (GWA) studies of malaria

Recent interest in identifying host genetic factors impacting on malaria has focused on severe malaria in African children using the genomewide association (GWA) study approach. Two signals near well-known malaria protective variants in *HBB* and *ABO* were detected in a meta-analysis including 5,425 cases [1], while a previous GWA study did not identify any variants exceeding the genomewide threshold until after the causal sickle cell trait mutation itself (HbS) was genotyped, illustrating the difficulties of covering genetic variability in African populations [2]. A third GWA study conducted in a population from Ghana identified two novel susceptibility genes, *ATP2B4*, encoding a red blood cell calcium pump, and *MARVELD3*, implicated in vascular adherence of infected red blood cells [3]. A reappraisal of GWA study data according to specific severe malaria subtype revealed opposing effects for the main African mutation underlying G6PDH deficiency: for severe anemia, a risk effect was observed, and for cerebral malaria, a protective effect, showing that phenotypic heterogeneity had previously masked this association. Numerous other genes previously validated under a candidate gene approach were missed by these same GWA studies, suggesting presence of further phenotypic heterogeneity. In addition to mutations underlying the hemoglobinopathies, notably the sickle cell trait and the thalessemias, genes previously identified for severe malaria using the candidate gene approach include those involved in: cytoadherence of infected red blood cells to the endothelium (*CD36*), antigen recognition (*HLA-B*), antibody response (*IL4*) and the proinflammatory response (*NOS2A*) (reviewed in [4]). The paucity of novel findings for severe malaria in spite of large sample sizes in past GWA studies suggests that phenotypic heterogeneity needs to be addressed, and that other malaria phenotypes merit further exploration, including parasitemia and IgG levels.

### The genetic basis for malaria infection phenotypes

Population-level observations, familial aggregation, and complex segregation analyses have established the genetic basis of parasitemia levels and IgG levels, providing the groundwork for the identification of novel protective or susceptibility variants. Parasitemia as a quantitative trait is usually an aggregate measure per individual from a longitudinal study (typically the mean or the maximum across values), with each measure defined as a density per uL of whole blood or as a percentage of red blood cells positive for trophozoites. Studies have generally focused on *P. falciparum* parasitemia. Mean parasitemia levels were found to differ between ethnicities based on studies conducted in Burkina Faso, Mali and Nepal for communities living in the same geographic area with similar levels of exposure to *P. falciparum* (reviewed in [4]). With respect to IgG levels, twin studies have revealed higher concordance for monozygotic than dizygotic twins for antibody response to specific parasite antigens, typically vaccine candidates [4]. Sibling correlation coefficients have been estimated at 0.16 to 0.24 and for IgG levels and at 0.13 to 0.39 for sub-type specific IgG levels [4]. Segregation analyses have led to a conclusion of complex genetic control for both parasitemia and IgG levels based on populations from Cameroon, Burkina Faso, and Papua New Guinea [4].

Heritability provides a measure of the human genetic contribution (proportion of variance) to a phenotype. Several estimates have been made addressing parasitemia during clinical malaria attacks and during an asymptomatic infection. In two communities from Senegal, one holoendemic for malaria and another with seasonal transmission (mesoendemic), a heritability of 0.22 was obtained for mean parasitemia during clinical attacks in both communities. A significant heritability of 0.13 for maximum parasitemia was obtained for mesoendemic transmission only. A maximum asymptomatic parasitemia heritability estimate of 0.22 was obtained for both communities, and a significant heritability of 0.33 for mean asymptomatic parasitemia was obtained for the endemic community [5]. In a hypoendemic region of Thailand characterized by co-infection with both *P. falciparum* and *P. vivax* malaria, mean and maximum trophozoite densities during clinical episodes displayed similar heritabilities to those obtained in Senegal for both *P. falciparum* (0.16 and 0.16) and *P. vivax* (0.13 and 0.15) [6]. In an exploration of risk factors of potential human infectiousness to mosquitoes (carriage of gametocyte stage parasites), there was significant heritability of *P. falciparum* gametocyte prevalence (0.15 - 0.57) for asymptomatic infections, but not during clinical attacks [7]. There is thus consistent evidence for human genetics contributing to a significant proportion of the observed variance in asexual and sexual parasite phenotypes with differences according to endemicity, parasite species, and whether the infection was symptomatic or not.

Genome-wide linkage studies have been performed on various study populations leading to the identification of loci controlling parasitemia. Chromosomal region 5q31-q33, containing several cytokine genes involved in Th1/Th2 balance, was linked to parasitemia among families in Cameroon [8] and in two independent study populations from Burkino Faso (reviewed in [4]), and more specifically to asymptomatic parasitemia in Burkina Faso [4] and Senegal [5]. The analogous mouse model region has also been identified for mouse malaria phenotypes [4]. The MHC region was linked to mild malaria in several studies, with chromosome 6p21 linked to asymptomatic parasitemia (reviewed in [4]). Other loci for the two related phenotypes, parasitemia (asymptomatic or during a clinical attack) and mild malaria, have been identified at 1p36, 2p25, 4q13-q21, 5p15-p13, 6p25.1, 6q15-q16, 9q34, 12q21-q22, 13q13, 10p15, 17p12, 19p13.12, 20p12 and 20q11 (reviewed in [4]).

### Parasitemia as a quantitative endophenotype

**Figure 1:**
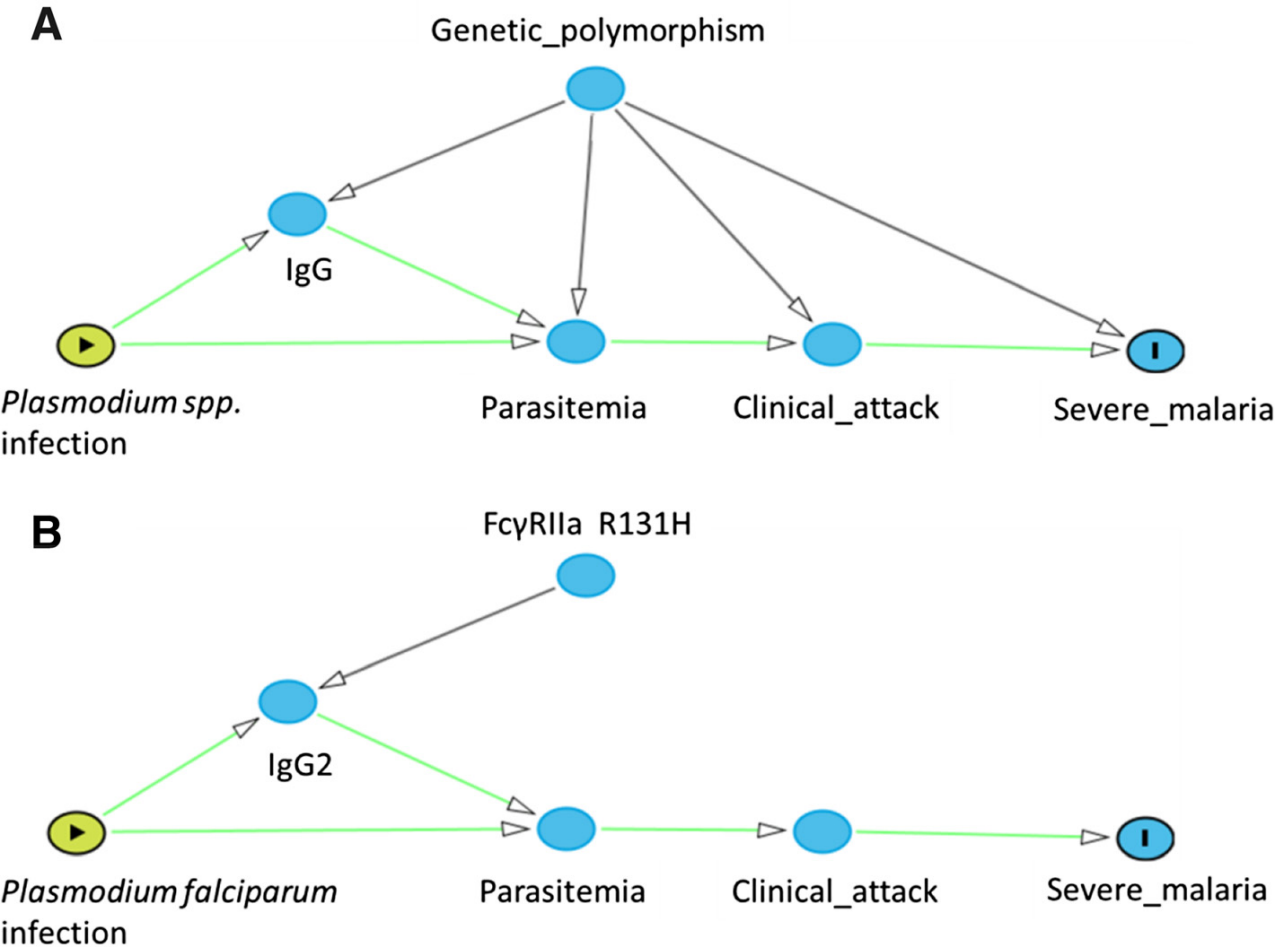
**Causal diagrams going from** ***Plasmodium spp.*** **infection to severe malaria through parasitemia and clinical attack.** **A**. Two possible pathways going from infection to parasitemia, via IgG-mediated acquired immunity or directly from infection to parasitemia, and multiple possible pathways for the impact of genetic polymorphism on IgG, parasitemia, clinical attack or severe malaria. **B**. An example specifying *Plasmodium falciparum* as the infective species, and the influence of genetic polyporphism at FcγRIIa distinguishing between FcγRIIa homozygotes for H131 vs. heterozygotes & homozygotes for R131 (recessive model) acting on parasitemia via IgG2 levels. The causal diagrams were created using DAGittyv.2.0 (http://www.dagitty.net/).

Parasitemia can be considered to be the result of two opposing forces in a tug-of-war, the pressure exerted by the malarial parasite in its multiplicative red blood cell stage, versus the pressure of anti-malarial immunity. The higher the parasitemia, the greater the malarial force, and the lower the force of anti-malarial immunity. For the study of human genetics, it would be optimal to focus on a measure of parasitemia that represents the global effectiveness of anti-malarial host immunity, which would assume factors contributing to malarial parasite pressure to be homogeneous across the study population, including parasite species and strain, and that all other parameters impacting on parasitemia, such as the initial parasite dose, are homogeneous or controlled for. Also, factors such as nutritional status, which affect host immunity without reflecting innate effectiveness should be constant in the population. These assumptions are met in part in the longitudinal study design that is limited geographically with contained vectorial transmission, and focused on a community with a similar lifestyle and socio-economic status as in a longitudinal study following an endemic community in Senegal for 22 years [9]. Host immunity is also known to vary with age and sex, which can be adjusted for. Under such homogeneous, controlled conditions, causal diagrams can offer a heuristic framework with which to evaluate potential causal pathways, highlighting genetic factors of interest (see Figure 1A) [10]. A progression is implied, going from the initial infection with *Plasmodium spp.* to the development of the infection through liver and blood-stage multiplication cycles (parasitemia as a quantitative trait), to clinical malaria attack, and to severe malaria. The initial infection stimulates the immune system, elicits protective IgG antibodies that limit parasitemia levels and represent the humoral aspect of anti-malarial host immunity. The arrows imply a causal relationship but do not imply a particular direction (increase or decrease). Arrows point from genetic polymorphism to all malaria phenotypes, indicating that all hypotheses are possible until tested, and thus the causal framework can be thought of as summarizing the working hypotheses which can then be reduced and refined as study results are obtained. Parasitemia occurs early in the causal pathway, prior to clinical symptoms and can be considered a malaria endophenotype. Genetic association studies evaluating specific polymorphisms can be tested for association with any of the measurable phenotypes in the causal progression, including parasitemia and IgG levels. Previous parasitemia candidate gene studies will be reviewed with examples considered in the context of this causal framework for the complex scenarios involving mediation through IgG levels. The candidate gene studies of parasitemia involve evaluation of the causal arrow from genetic polymorphism to parasitemia.

The first genetic association studies with malaria parasitemia levels began with the observation of the co-occurrence of geographic regions of high malaria prevalence and high prevalence of certain hemoglobinopathies [11]. Hemoglobinopathies belong either to the category of structurally variant forms of hemoglobin (such as sickle cell trait) or the thalassaemias, resulting in reduced or absent production of molecules forming normal adult hemoglobin A. Allison first reported a major role for the sickle cell mutation (HbS) in the β-globin gene in the protection against *P. falciparum* parasitemia as a binary trait in 1954 [12]. He also noted that subjects with HbS displayed a lower proportion of moderate parasitemia and none displayed severe parasitemia compared to homozygous HbA individuals [12]. Genetic association studies of the hemoglobinopathy variants with mild malaria phenotypes have recently been compiled in a systematic review and meta-analysis [13], where parasitemia was considered as a binary trait in a cross-sectional design and compared to healthy controls. Neither HbS nor the thalassemia variants were found to protect against parasitemia. Studies evaluating parasitemia quantitatively and longitudinally have found contrasting results for the evidence of the impact of HbS heterozygotes on parasite density, with some observations of lower levels compared to HbA homozygotes [14], others displaying similar levels [15] or even higher levels [16]. Given the contrasting results, in the context of the literature, it appears that the casual arrow from HbS to parasitemia holds, such that in part, parasitemia is a mediator of the association leading to clinical malaria, and the arrows from polymorphism directly to clinical attack and severe malaria also hold, such that part of the association with clinical malaria phenotypes is independent of parasitemia (Figure 1A).

Genetic association studies of parasitemia as a quantitative trait have been conducted using a candidate gene approach, focused on immune signaling genes or to evaluate whether parasitemia mediates previously discovered associations with clinical malaria. ABO blood group has been shown to impact on clinical malaria risk with group O in *P. falciparum* malaria-endemic regions displaying a protective effect. Following from this observation, lowest mean parasitemia was observed for group O compared to A, B or AB groups consistently across studies (reviewed in [17]). Genetic disorders of the red blood cell membrane, including hereditary spherocytosis and southeast Asian ovalocytosis, have been associated with resistance to malaria infection. Hereditary spherocytosis has been associated with lower parasitemia [18], while ovalocytosis has not revealed differential parasitemia levels (reviewed in [19]). Several immune response genes have been associated with parasitemia levels including toll-like receptor gene polymorphisms. *IL4*, *IL12B*, *LTA*, *NCR3* have either been linked to mild malaria or parasitemia, alleles in these genes have been associated with these phenotypes (reviewed in [20]). Promoter polymorphisms in *TNF* have been associated with lower levels of parasitemia [21]. In a family-based study from Burkina Faso, significant interactions impacting on parasitemia levels were identified involving *HBB* variant HbC and *LTA* + 80 or *IL12B* polymorphisms [22]. In a linkage study conducted in a Senegalese study population followed up by family-based association tests within linked regions, a polymorphism in *ARHGAP26* was found to be associated with intensity of plasmodial infection in the 5q33.1 region [23]. Interestingly, a hypothesis-free approach led to the identification of a compelling candidate gene: the protein encoded by *ARHGAP26* is implicated in regulation of Rho family signal transduction of endothelial receptors of *P. falciparum* infected red blood cells [23]. Other pathways have also been explored such as the heparan sulphate biosynthesis pathway [24]. These studies were conducted using a hypothesis-driven approach and provide a proof of principle of the potential for parasitemia as a phenotype to reveal genetic associations. Thus, for some of these polymorphisms, parasitemia appears to be the mediator of associations with clinical malaria phenotypes, while for others the associations with clinical malaria phenotypes are independent of parasitemia. Therefore, parasitemia may also enable the discovery of new associations using hypothesis-generating GWA approaches.

### The functional imperative and IgG levels

A key potential mediator of anti-malarial host immunity is IgG: passive transfer of IgG, purified from sera of semi-immune adults to non-immune patients resulted in clearance of parasitemia [25]. The potential to clear parasitemia depends upon IgG subtype. Specifically, immunoglobulins IgG1 and/or IgG3 bind with high affinity to Fc receptors on phagocytic cells, thereby activating effector mechanisms, while IgG2 and IgG4 bind with lower affinity (reviewed in [25]). Numerous epidemiological studies have investigated IgG levels by subtype, antigen specificity and level of antibody production to determine the features most important in determining clinical immunity with consideration for seasonality [26]. Studies identifying IgG levels or subtype-specific IgG levels as mediators of association between genetic polymorphisms and malaria phenotypes provide a clear framework for the functional hypothesis connecting the polymorphism to malaria, implicating IgG synthesis molecular pathways.

Several studies have investigated the role of IgG levels as a mediator of *HBB* polymorphism on parasitemia levels. For example, a study in a population in Burkina Faso with a frequency of 0.14 of the C beta-globin allele (hemoglobin C, HbC), and a low frequency of the S allele, showed reduced maximum *P. falciparum* parasitemia levels among HbC subjects [27]. HbC was positively associated with anti-malarial IgG levels in the same population, suggesting that *HBB* polymorphisms alter anti-malarial IgG production [20]. HbS has also been tested for association with IgG subclasses in Gabon [28], and, adding another layer of specificity, another study looked at IgG responses to the parasite’s major cytoadherence ligand, *P. falciparum* erythrocyte membrane protein 1 (PfEMP1). Complex causal hypotheses (Figure 1A) can thus be constructed based on these results, with *Plasmodium* species specificity, IgG sub-type and antigen specificity that would require validation in subsequent studies, and complement the studies mentioned above focused on parasitemia only. Similar studies investigating the role of IgG levels have been conducted for other polymorphisms associated with parasitemia levels, for example the protective effect of TNF promoter polymorphisms may be partly due to their effect on IgG subclass production [20], and an association of anti-malarial IgG levels with IL4-590 has been reported [29].

Protective immunity in malaria is at least in part transferred through antibodies, and the probable modes are through inhibition of merozoite invasion of erythrocytes and/or a role for Ab/complement mediated phagocytosis via Fc-receptors (FcRs), although which of the large family of human FcRs are optimally involved remains unclear [30]. Important examples include receptors for IgG (FcγRI, FcγRII, FcγRIII, FcRn), IgE (FcɛRI), IgA (FcαRI, Fcα/μR, pIgR) and IgM (Fcα/μR) [30]. Several association studies have focused on *FCGR2A* that encodes the human IgG receptor FcγRIIa [30]. Although IgG2 generally binds Fc receptors with low affinity, IgG2 levels were associated with a protective effect against malaria among individuals homozygous for the *FCGR2A* H131 variant, which influences binding of IgG2 on neutrophils [31]. A significant association of the FcγRIIa H131 allele with IgG2 levels, based on a recessive model, was reported in a family based study [20] and in independent population based studies [32,33]. Thus, IgG2 appears to be a mediator of the effect of H131 homozygotes leading to a protective effect on malaria clinical disease [34]. The specific causal pathway for this association is represented in Figure 1B. The polymorphism FcγRIIa R131H acts on parasitemia via IgG2 levels as a mediator (Figure 1B) from genetic polymorphism to IgG2.

Carriers of Glucose-6-phosphate dehydrogenase deficiency (G6PD deficiency) alleles are partially protected against malaria. In a study of children in an area with seasonal malaria transmission in Senegal, *G6PD* A- carriers had a lower increase of IgG3 levels to merozoite surface antigens during the transmission season than the non-carriers, which is consistent with carriers having a lower burden of merozoites, and indicative that protective immunity is likely to occur through other mechanisms than through humoral immunity. Such mechanisms could be cell-mediated immunity and/or physiological consequences of the blood disorder could explain clinical protection [35]. In a study specifically investigating the impact of the mutation *G6PD-*Mahidol (487A) on a population infected with both *P. falciparum* and *P. vivax*, the variant form was associated with protection from high *P. vivax* parasite density, but not high *P. falciparum* parasite density in humans, indicating *Plasmodium spp.* specificity for the protective effect [36]. Along with *G6PD* deficiency leading to protective or susceptibility effects depending on specific severe malaria phenotype as mentioned above, collectively, these studies demonstrate the causal relationship from *G6PD* deficiency to IgG subtype to parasite species specificity, to parasitemia levels and clinical malaria specificity. Population level longitudinal studies focused on quantitative traits, such as parasitemia levels and IgG subtype levels, and specification of the severe malaria phenotype, enabled the identification of genetic associations that also provide insight into the functional relationship between the polymorphisms at this gene and disease.

## Conclusions

Although to date, the main phenotypic focus of studies on the human genetics of malaria have been on severe malaria, we show here, through key examples, that malaria infection, viewed as a collection of related quantitative traits, particularly parasitemia and sub-type specific IgG levels, has high potential to increase understanding of the genetic architecture of malaria. Studies focusing on these phenotypes act as a bridge between genetic association studies and functional studies, and enable the elaboration and verification of highly specific hypotheses (Figure 1A). Combinations of particular parasite species, phenotypes and genetic polymorphism genotypes may be linked causally. A recent focus on severe malaria only has been demonstrated to be short-sighted given the paucity of new findings to come from GWA studies. Looking to future studies, there is clearly potential for further exploiting the information available in longitudinal studies that have measured parasitemia at regular intervals by considering the rate of change in parasitemia levels as well as absolute levels. This is especially true for malaria, where the acquisition of clinical immunity develops only after repeated clinical episodes and completely sterilizing immunity is never achieved. A longitudinal GWA study could thus reveal novel genetic variants controlling parasitemia levels. Regarding the study of IgG levels, most studies either evaluate total levels by subtype or focus on response to a single antigen at a time. Multivariate statistical methods considering IgG levels against a panel of relevant antigens IgG simultaneously, adapted to GWA studies are needed to optimally capture relevant response. Furthermore, genomics approaches could also be applied to all *Plasmodium spp.* to more fully account for pathogen variability.
